# The incidence of penetrating trauma in London: have previously reported increases persisted in the last six years?

**DOI:** 10.1186/1757-7241-22-S1-P3

**Published:** 2014-07-07

**Authors:** Kate Crewdson, Anne E Weaver, Gareth E Davies, David J Lockey

**Affiliations:** 1 London’s Air Ambulance, Royal London Hospital, London, E1 1BB, UK

## Background

We have previously reported a 20.5% annual rise in penetrating trauma attended by our pre-hospital trauma service between 1991 and 2006. Data from national registries was conflicting and surprisingly difficult to obtain. We established an increasing trend and predicted a continued rise. This study was performed to establish whether this prediction was correct. Rising penetrating trauma rates have implications for emergency medical care provision and policing.

## Method

A retrospective review of the London Air Ambulance database was conducted to identify patients who sustained deliberate penetrating trauma from January 1^st^ 2007 to December 31^st^ 2012. Children, and patients who died in the pre-hospital phase were included. The number of pre-hospital resuscitative thoracotomies performed by the service was also recorded.

## Results

The median number of penetrating trauma cases attended per year was 95, (range 14-220) in the period 1991-2006, and 352.5, (range 205-416) from 2007 to 2012. These figures represent 9.9% and 19.3% of the annual trauma caseload respectively. The mean increase per annum was 20.5% in the initial study, which equates to a mean increase of 10.3 cases per year (R = 0.89) over the 16 years, and 14% in the follow-up study, equivalent to 32.7 cases per year (R = 0.81). The corresponding increase in blunt trauma of 1.9%, 30.8 cases per year (R = 0.86) from 1991-2006, and 8.9% per year or 75.2 cases / year (R=0.84) between 2007-2012, was less than that observed for penetrating trauma (p < 0.0001).

There was a corresponding rise in the number of pre-hospital thoracotomies performed in the same period.

## Conclusion

The rise in penetrating trauma that we reported in our pre-hospital trauma service in the period 1991-2006 has continued to rise in the period 2007-2013. This has considerable impact on the provision of trauma services and policing.

